# In Silico and Experimental Evidence for Theasaponin E2 as a Modulator of the AKT1/GSK-3β Signaling Axis in Osteoclast Differentiation

**DOI:** 10.3390/ijms27209255

**Published:** 2026-10-18

**Authors:** Lifang Zhang, Yili Ding, Xinyi Bao, Vishwa Deepak

**Affiliations:** 1 Osteoimmunology & Drug Discovery Research Group, Department of Biology, College of Science, Mathematics and Technology, Wenzhou-Kean University, 88 Daxue Road, Wenzhou 325060, China; 2 Dorothy and George Hennings College of Science, Mathematics and Technology, Kean University, 1000 Morris Ave, Union, NJ 07083, USA; yding@kean.edu; 3 Department of Chemistry, College of Science, Mathematics and Technology, Wenzhou-Kean University, 88 Daxue Road, Wenzhou 325060, China; 4 International Frontier Interdisciplinary Research Institute (IFIRI) of Wenzhou-Kean University, Wenzhou 325060, China

**Keywords:** theasaponin E2, osteoclastogenesis, RANKL, AKT/GSK-3β signaling, molecular docking, bone resorption

## Abstract

Theasaponin E2 (TE2) is a triterpenoid saponin isolated from Camellia sinensis seeds with reported anti-inflammatory, antibacterial, and antitumor activities; however, its effects on osteoclasts remain unclear. In this study, TE2 markedly inhibited RANKL-induced osteoclast differentiation in RAW264.7 cells without affecting precursor-cell viability. TE2 also significantly reduced the number and viability of mature, differentiated osteoclasts. Mechanistically, TE2 suppressed RANKL-induced phosphorylation of AKT at Ser473 and GSK-3β at Ser9, as determined by ELISA. Molecular docking predicted that TE2 could interact with the interdomain allosteric pocket of AKT1 at the pleckstrin homology–kinase domain interface (PDB ID: 7NH5; docking score, −9.8 kcal/mol) and the ATP-binding pocket of GSK-3β (PDB ID: 5K5N; docking score, −7.7 kcal/mol). The selected docking poses contained multiple hydrogen-bonding interactions with donor–acceptor distances of 2.79–3.19 Å. Collectively, these findings indicate that TE2 suppresses osteoclast differentiation and reduces mature osteoclast viability, potentially through modulation of the AKT/GSK-3β signaling axis. TE2 may therefore represent a promising natural-product candidate for further investigation in conditions characterized by excessive osteoclast-mediated bone resorption.

## 1. Introduction

Osteoclasts are multinucleated cells primarily responsible for bone resorption [1]. Their differentiation from monocyte/macrophage-lineage precursors is regulated principally by receptor activator of nuclear factor-κB ligand (RANKL) and macrophage colony-stimulating factor (M-CSF) [2]. Binding of RANKL to its receptor, RANK, activates multiple downstream signaling pathways, including the phosphoinositide 3-kinase/AKT pathway [3]. Activated AKT phosphorylates glycogen synthase kinase-3β (GSK-3β) at Ser9, thereby suppressing its activity and promoting the nuclear accumulation and transcriptional activity of nuclear factor of activated T cells c1 (NFATc1), the master regulator of osteoclast differentiation [3,4]. Excessive osteoclast formation and activity contribute to pathological bone resorption in disorders such as osteoporosis, periodontitis, and inflammatory bone loss [5]. Therefore, identifying compounds that inhibit osteoclastogenesis by modulating the AKT/GSK-3β signaling axis may support the development of new therapeutic strategies for osteolytic diseases.

AKT and GSK-3β occupy a functionally linked position within this cascade: AKT-mediated Ser9 phosphorylation of GSK-3β is required to relieve GSK-3β-dependent restraint of NFATc1, while GSK-3β itself can independently regulate NFATc1 nuclear localization and stability [6]. Because these kinases constitute functionally connected signaling nodes that regulate NFATc1 activity, their coordinated modulation may influence osteoclast differentiation. This rationale motivated our evaluation of theasaponin E2 (TE2) as a candidate capable of engaging both nodes of the AKT/GSK-3β axis.

Natural products remain an important source of lead compounds for regulating kinase-dependent signaling pathways because of their considerable structural and chemical diversity [7]. Theasaponin E2 (TE2) is a triterpenoid saponin isolated from the seeds of Camellia sinensis [8]. Structurally, TE2 belongs to the barringtogenol C-type oleanane triterpenoid saponins and contains an acylated aglycone core linked to an oligosaccharide chain. Tea saponins have also been reported to modulate kinase-associated signaling pathways, including MAPK/ERK signaling, in other cellular systems [9,10]. However, the effects of TE2 on osteoclast differentiation and survival have not been characterized, and its potential interactions with kinases involved in osteoclast signaling remain unknown.

In this study, we investigated the effects of TE2 on RANKL-induced osteoclast differentiation and mature osteoclast viability in vitro. We further examined its effects on AKT and GSK-3β phosphorylation and performed molecular docking analyses to explore potential interactions of TE2 with both kinases.

## 2. Results

### 2.1. TE2 Suppresses RANKL-Induced Osteoclast Differentiation Without Affecting Precursor Viability

**Figure 1:**
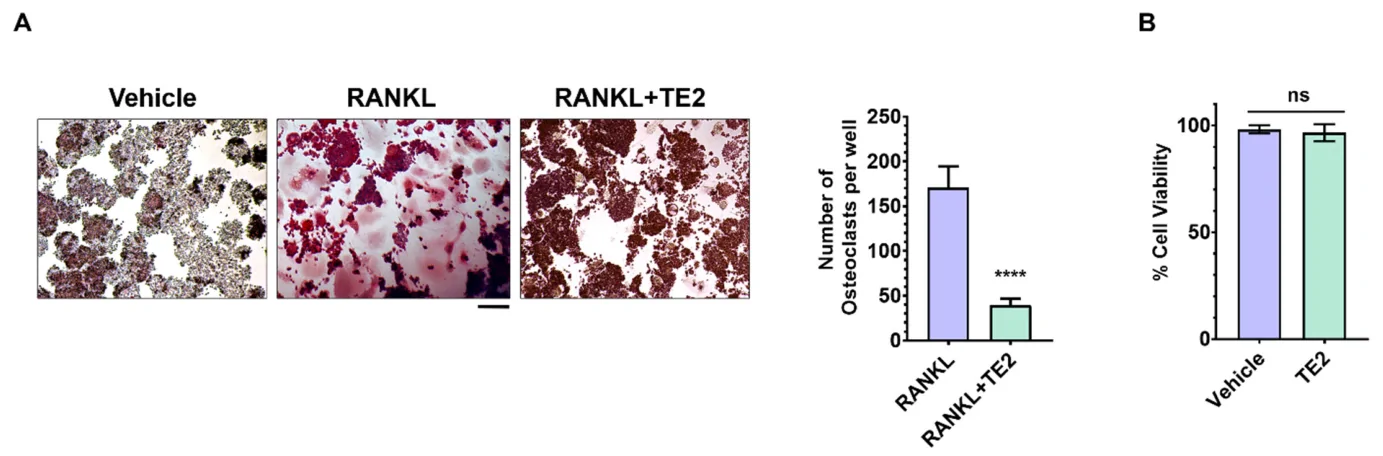
Theasaponin E2 (TE2) inhibits RANKL-induced osteoclast differentiation in RAW264.7 cells. (**A**) RAW264.7 cells were cultured with RANKL (50 ng/mL) in the presence or absence of TE2 (1 μM) for 5 days. Cells were fixed and stained for tartrate-resistant acid phosphatase (TRAP) to visualize mature osteoclasts. Representative images of the vehicle, RANKL, and RANKL + TE2 groups are shown. TRAP-positive multinucleated cells containing three or more nuclei were counted as osteoclasts. The bar graph shows the number of osteoclasts per well. Scale bar, 100 μm. (**B**) RAW264.7 cells were treated with vehicle or TE2 (1 μM) for 48 h, and cell viability was assessed using the CCK-8 assay. Data are presented as the mean ± SD from three independent experiments (*n* = 3). Statistical significance was determined using an unpaired, two-tailed Student’s *t*-test. **** *p* < 0.0001 versus the RANKL-treated group; ns, not significant versus the vehicle group.

We first examined the effect of TE2 on RANKL-induced osteoclastogenesis in RAW264.7 cells. TRAP staining showed that TE2 treatment (1 μM) markedly reduced the formation of TRAP-positive multinucleated osteoclasts compared with RANKL treatment alone (Figure 1A). In contrast, TE2 did not significantly affect the viability of RAW264.7 osteoclast precursor cells (Figure 1B), indicating that its inhibitory effect on osteoclast differentiation was not attributable to nonspecific cytotoxicity.

### 2.2. TE2 Suppresses AKT/GSK-3β Signaling and Impairs Mature Osteoclast Survival

**Figure 2:**
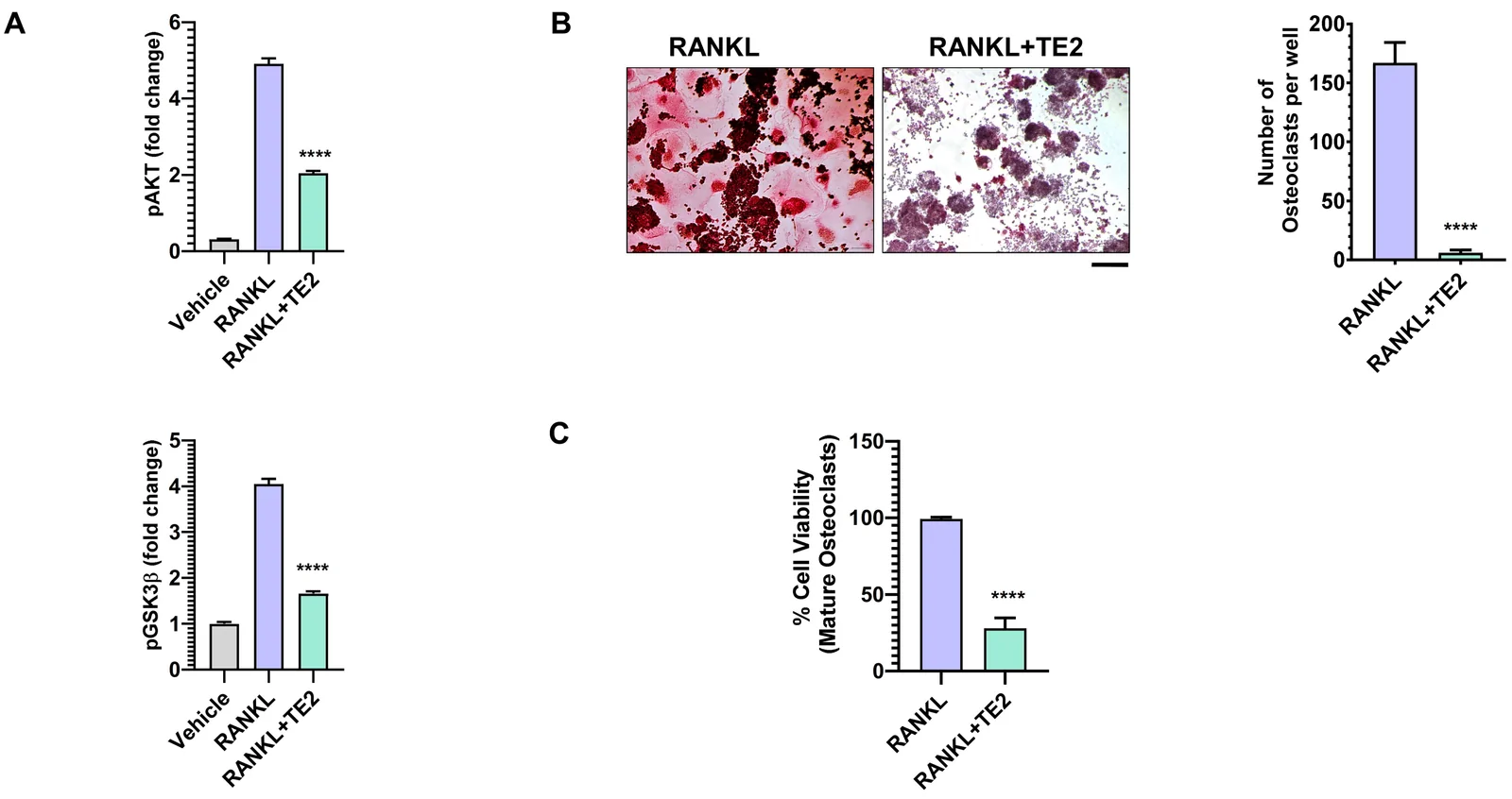
Theasaponin E2 (TE2) suppresses RANKL-induced AKT/GSK-3β phosphorylation and reduces the number and viability of mature osteoclasts. (**A**) RAW264.7 cells were pretreated with TE2 (1 μM) for 1 h and subsequently stimulated with RANKL (50 ng/mL) for 10 min. The phosphorylation levels of AKT at Ser473 and GSK-3β at Ser9 were measured by ELISA and expressed as fold changes relative to the vehicle-treated group. (**B**) RAW264.7 cells were differentiated into mature osteoclasts by treatment with RANKL for 5 days and subsequently treated with vehicle or TE2 (1 μM) for 24 h. Representative TRAP-stained images of the RANKL and RANKL + TE2 groups are shown. TRAP-positive multinucleated cells containing three or more nuclei were counted as osteoclasts, and the graph shows the number of osteoclasts per well. Scale bar, 100 μm. (**C**) The viability of mature osteoclast cultures following treatment with TE2 (1 μM) for 24 h was assessed using the CCK-8 assay. Data are presented as the mean ± SD from three independent experiments (*n* = 3). Panel A was analyzed using one-way ANOVA followed by Tukey’s multiple-comparisons test, whereas panels B and C were analyzed using an unpaired, two-tailed Student’s *t*-test. **** *p* < 0.0001 versus the RANKL-treated group.

To investigate the signaling mechanism underlying the inhibitory effect of TE2, we examined RANKL-induced phosphorylation of AKT and GSK-3β. ELISA analysis showed that TE2 significantly reduced the phosphorylation of AKT at Ser473 and GSK-3β at Ser9 (Figure 2A). These findings indicate that TE2 modulates RANKL-induced AKT/GSK-3β signaling by reducing AKT Ser473 and GSK-3β Ser9 phosphorylation.

We next evaluated the effect of TE2 on differentiated osteoclasts. RAW264.7 cells were differentiated with RANKL for 5 days and subsequently treated with TE2 for 24 h. TRAP staining revealed a marked reduction in the number of mature osteoclasts following TE2 treatment (Figure 2B). Consistently, the CCK-8 assay showed a significant reduction in the viability of mature osteoclast cultures (Figure 2C). These results suggest that mature osteoclasts are more susceptible to TE2 treatment than undifferentiated RAW264.7 precursor cells under the conditions tested.

### 2.3. Molecular Docking Predicts Potential Interactions of TE2 with AKT1 and GSK-3β

To examine whether TE2 could potentially interact with AKT1 and GSK-3β, molecular docking analyses were performed. TE2 was predicted to occupy the allosteric pocket at the interface between the pleckstrin homology and kinase domains of AKT1 (PDB ID: 7NH5), with a predicted docking score of −9.8 kcal/mol. The selected pose formed hydrogen bonds with Tyr18A, Asp274A, Glu278A, and Leu156A, with donor–acceptor distances ranging from 2.79 to 3.19 Å, together with hydrophobic contacts involving Lys297A, Gly311A, and Cys310A.

**Figure 3:**
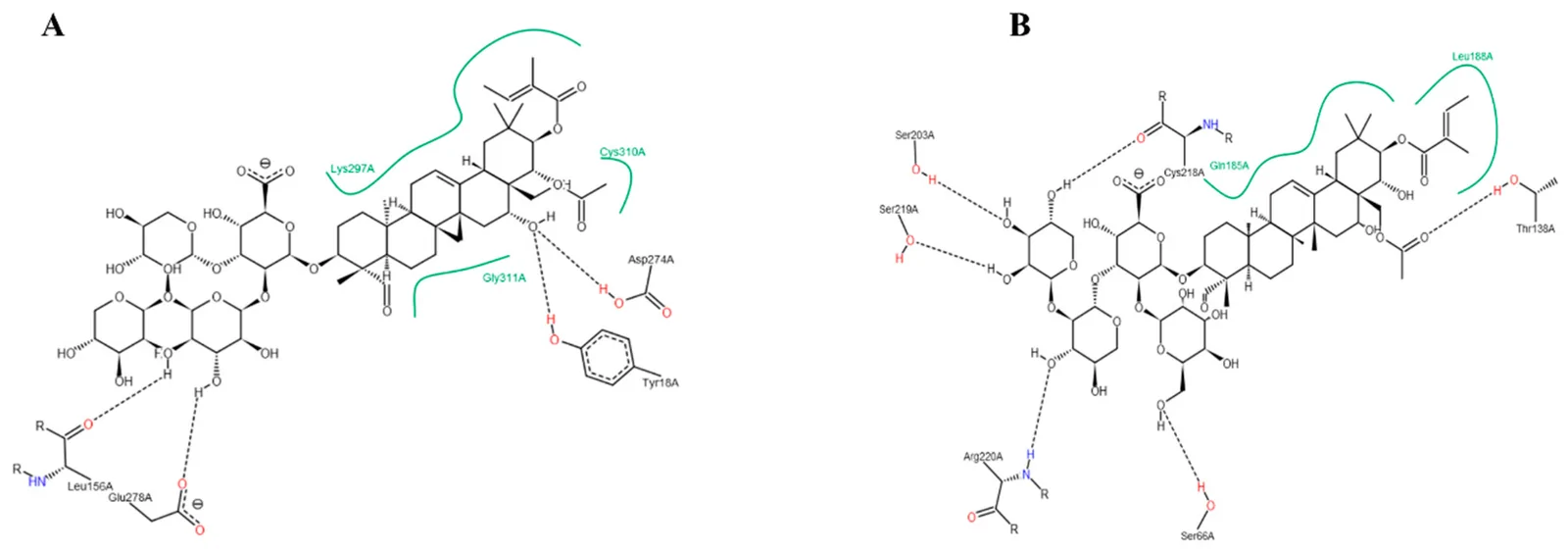
Predicted molecular interactions of theasaponin E2 (TE2) with AKT1 and GSK-3β. Two-dimensional interaction diagrams of the selected docking poses of TE2 with (**A**) AKT1 (PDB ID: 7NH5; predicted binding energy, −9.8 kcal/mol) and (**B**) GSK-3β (PDB ID: 5K5N; predicted binding energy, −7.7 kcal/mol). Molecular docking was performed using CB-Dock3, and protein–ligand interaction diagrams were generated using PoseView [11]. In AKT1, TE2 was predicted to bind within the interdomain allosteric pocket between the pleckstrin homology and kinase domains, forming hydrogen bonds with Tyr18A, Asp274A, Glu278A, and Leu156A, and hydrophobic contacts with Lys297A, Gly311A, and Cys310A. The corresponding hydrogen-bond distances were 2.86, 2.79, 2.97, and 3.19 Å, respectively. In GSK-3β, TE2 was predicted to bind within the ATP-binding pocket, forming hydrogen bonds with Thr138A, Ser203A, Arg220A, Ser66A, Cys218A, and Ser219A, and hydrophobic contacts with Gln185A and Leu188A. The corresponding hydrogen-bond distances were 2.82, 2.90, 2.95, 3.00, 2.99, and 3.06 Å, respectively. Hydrogen bonds were defined using a donor–acceptor distance cutoff of <3.5 Å. Dashed lines indicate hydrogen bonds, solid green lines indicate hydrophobic contact regions, and R groups represent continuation of the protein backbone beyond the depicted residues.

TE2 was also predicted to bind within the ATP-binding pocket of GSK-3β (PDB ID: 5K5N), with a predicted docking score of −7.7 kcal/mol. The selected pose formed hydrogen bonds with Thr138A, Ser203A, Arg220A, Cys218A, Ser66A, and Ser219A, with distances ranging from 2.82 to 3.06 Å, and hydrophobic contacts with Gln185A and Leu188A (Figure 3 and Table 1). These computational findings provide a possible structural basis for the observed suppression of AKT and GSK-3β phosphorylation observed by ELISA.

## 3. Discussion

In this study, we demonstrate that theasaponin E2 (TE2) inhibits RANKL-induced osteoclast differentiation in vitro and reduces the viability of mature osteoclasts. The AKT signaling pathway plays an important role in osteoclast differentiation and survival. Inhibition of AKT phosphorylation decreases the inhibitory phosphorylation of GSK-3β at Ser9, thereby maintaining GSK-3β activity and suppressing NFATc1-dependent osteoclastogenesis [12,13]. Accordingly, the reductions in both p-AKT and p-GSK-3β observed in the present study indicate that TE2 interferes with the AKT/GSK-3β signaling axis.

These findings extend a growing body of work identifying natural products as modulators of osteoclastogenic signaling. We have previously reported that another plant-derived compound, 8-epixanthatin, suppresses RANKL-induced osteoclast differentiation via inhibition of NF-κB and MAPK signaling, underscoring that structurally diverse natural products can converge on distinct nodes of the osteoclast differentiation program [14]. TE2 adds to this landscape as, to our knowledge, the first triterpenoid saponin reported to engage the AKT/GSK-3β axis specifically in osteoclasts, complementing prior reports of tea-saponin modulation of MAPK/ERK signaling in other cell systems and echoing findings with other natural compounds, such as fucoidan, that similarly act through the Akt/GSK-3β/NFATc1 cascade to suppress osteoclastogenesis [15], suggesting that this compound class may act through partially overlapping but pathway-specific mechanisms depending on cellular context. The convergence of AKT/GSK-3β signaling on NFATc1 activation [6], a node targeted by several existing antiresorptive strategies, is what makes this axis an attractive target: compounds acting here have the potential to intercept osteoclast differentiation upstream of the terminal transcriptional program, rather than only at later stages of resorptive activity.

Molecular docking provided a possible structural basis for these functional effects. TE2 was predicted to occupy the interdomain allosteric pocket of AKT1 and the ATP-binding pocket of GSK-3β, with favorable docking scores and multiple hydrogen-bonding interactions at donor–acceptor distances of 2.79–3.19 Å. The predicted AKT1-binding pose was located within the allosteric pocket represented in structure 7NH5 [16], whereas the GSK-3β pose was located within the ATP-binding region represented in structure 5K5N [17]. These findings raise the possibility that TE2 interacts with both kinases and modulates the AKT/GSK-3β signaling axis through a dual-target mechanism. However, because docking scores and predicted interaction patterns do not establish direct binding or biochemical inhibition, this interpretation requires experimental confirmation.

TE2 also produced different effects in precursor and mature osteoclast cultures. At the concentration tested, TE2 suppressed RANKL-induced osteoclast differentiation without reducing the viability of RAW264.7 precursor cells, whereas treatment of differentiated osteoclasts markedly decreased both osteoclast number and culture viability. These findings suggest that the differential response may reflect the dependence of mature osteoclast survival on sustained prosurvival signaling, including the PI3K/AKT pathway, or other stage-specific vulnerabilities [18]. This response is potentially relevant to pathological bone resorption because a compound that inhibits the formation of new osteoclasts while reducing the persistence of existing mature osteoclasts could exert complementary antiresorptive effects. Nevertheless, additional experiments in primary osteoclast precursors, mature osteoclasts, and other myeloid-lineage cells are required before conclusions regarding cell-type selectivity can be drawn.

TE2 was selected for detailed characterization on the basis of an efficient upstream drug-screening strategy using a HEK293-RANK-NF-κB-luciferase reporter system, in which TE2 emerged as a strong candidate for suppressing RANKL-responsive signaling among the compounds tested; at the concentration carried forward into the present study, TE2 did not affect RAW264.7 precursor viability (Figure 1B), arguing against generalized cytotoxicity as the basis for its effects on differentiation. We recognize that TE2 has previously been reported to possess anti-inflammatory, antibacterial, and antitumor activities, indicating that it may engage multiple cellular targets beyond AKT and GSK-3β. Such polypharmacology is a recognized feature of many triterpenoid saponins [19] and does not preclude a meaningful contribution of AKT/GSK-3β modulation to the osteoclast phenotypes observed here, but it does mean that some of TE2’s effects, particularly on mature osteoclast viability, could involve additional or off-target pathways not captured by the present signaling assays. Distinguishing AKT/GSK-3β-dependent from -independent contributions to TE2’s activity, for example, using pathway-selective rescue or knockdown approaches, is an important direction for future work.

Several limitations should be acknowledged. First, the molecular docking analysis is computational and hypothesis-generating and does not demonstrate direct physical binding of TE2 to AKT1 or GSK-3β. Direct target engagement should therefore be examined using orthogonal approaches such as surface plasmon resonance, isothermal titration calorimetry, cellular thermal shift assays, or related binding methods. Cell-free kinase assays are also required to determine whether TE2 directly inhibits AKT1, GSK-3β, or both enzymes. Molecular dynamics (MD) simulations to evaluate the temporal stability of the predicted TE2-AKT1 and TE2-GSK-3β complexes would further strengthen confidence in the docking-derived poses; such simulations, together with the orthogonal binding assays noted above, were considered beyond the intended scope of the present brief report, which is focused on establishing an initial, testable hypothesis for TE2’s mechanism of action, and are planned as a follow-up computational study. Second, the present study focused on p-AKT (Ser473) and p-GSK-3β (Ser9) as proximal readouts of the signaling axis under investigation; downstream transcriptional targets such as NFATc1 were not directly assessed here but represent a logical extension for future work aiming to fully trace this cascade to its functional endpoint. Third, the mechanism responsible for the reduced viability of mature osteoclasts remains unclear. Further studies should determine whether TE2 induces apoptosis, alters cytoskeletal organization, promotes osteoclast detachment, or affects other survival pathways. Finally, the present findings were obtained in a single in vitro RAW264.7-cell model, and the present study did not examine the duration of TE2’s suppressive effect on osteoclast differentiation or function beyond the timepoints tested. As a brief report, this study is intended to establish an initial, mechanistically motivated foundation rather than a comprehensive characterization; extending these observations to primary bone marrow macrophages and additional myeloid-lineage cell types, assessing the durability of TE2’s effects over extended culture periods, and validating findings in relevant animal models of osteoporosis or inflammatory bone loss will be important directions for future work. In particular, triterpenoid saponins as a class are frequently characterized by limited oral bioavailability and variable metabolic stability [19]; formal pharmacokinetic and toxicity studies of TE2 will therefore be necessary before its therapeutic potential can be established.

## 4. Materials and Methods

### 4.1. Compound Selection, Cell Culture, and Reagents

Theasaponin E2 (TE2) was selected from a prior single-concentration screen of natural-product candidates. Compounds were tested in HEK-293T cells (Wuhan Pricella Biotechnology Co., Ltd., Wuhan, China) stably expressing an NF-κB-responsive firefly luciferase reporter (NFκB-TA-Luc-EF1α-mCherry; Beyotime, Shanghai, China) and RANK (Mouse TNFRSF11A; Sino Biological, Beijing, China) (HEK/RANK-NF-κB-Luc). Primary screens of natural-product libraries are commonly performed at a single concentration, with detailed characterization reserved for the most promising hits [20]. TE2 strongly suppressed RANKL-responsive reporter activity and was advanced for detailed characterization in the osteoclast assays described below. Full concentration-response profiling of TE2 was outside the scope of the primary screen and is planned for a follow-up study.

Murine macrophage RAW264.7 cells (Wuhan Pricella Biotechnology Co., Ltd., Wuhan, China) were cultured in Dulbecco’s Modified Eagle Medium (DMEM; Gibco, Thermo Fisher Scientific, Waltham, MA, USA) supplemented with 10% fetal bovine serum and 1% penicillin–streptomycin at 37 °C in a humidified incubator with 5% CO_2_ as described earlier [14]. Theasaponin E2 (TE2) (purity ≥ 98.76%; CAS No.: 220114-30-7; HY-N12153) was purchased from MedChemExpress (Monmouth Junction, NJ, USA). Recombinant mouse RANKL (462-TEC-010) was obtained from R&D Systems (Minneapolis, MN, USA).

### 4.2. Cell Viability Assay

RAW264.7 cells were seeded in 96-well plates (5 × 10^3^ cells/well) and treated with vehicle or TE2 (1 μM) for 48 h. Cell viability was assessed using a Cell Counting Kit-8 (CCK-8; Service Bio, Wuhan, China), a colorimetric assay based on bioreduction of the water-soluble tetrazolium salt WST-8 by cellular dehydrogenases [21], according to the manufacturer’s protocol. Cell viability was expressed as a percentage relative to vehicle-treated controls.

### 4.3. Osteoclast Differentiation and Mature Osteoclast Treatment

To assess effects on osteoclast differentiation, RAW264.7 cells were seeded in 96-well plates (5 × 10^3^ cells/well) and assigned to three groups: vehicle-only, RANKL-only (50 ng/mL), or RANKL (50 ng/mL) plus TE2 (1 μM), and cultured for 5 days. The culture medium, containing the corresponding additive(s) for each group (vehicle, RANKL alone, or RANKL plus TE2), was refreshed every 2 days for all three groups. Cell morphology and attachment were monitored microscopically throughout the differentiation period. After differentiation, cells were fixed with 4% paraformaldehyde for 15 min and stained for tartrate-resistant acid phosphatase (TRAP) activity using a commercial TRAP staining kit (Servicebio, Wuhan, China) according to the manufacturer’s protocol. TRAP-positive multinucleated cells (≥3 nuclei) were counted as mature osteoclasts under light microscopy, a widely used criterion for identifying differentiated osteoclasts in vitro [14]. To assess effects on already-differentiated osteoclasts, in a separate set of experiments RAW264.7 cells were first differentiated into mature osteoclasts with RANKL alone for 5 days and subsequently treated with either vehicle or TE2 (1 μM) for 24 h; TRAP staining and cell viability (CCK-8 assay) were then assessed to evaluate osteoclast survival under this post-differentiation treatment paradigm.

### 4.4. Signaling Analysis

RAW264.7 cells were pre-treated with TE2 (1 μM) for 1 h prior to RANKL stimulation (50 ng/mL, 10 min), a stimulation window consistent with established protocols for capturing RANKL-induced AKT/GSK-3β phosphorylation dynamics in osteoclast precursors [12]. Phosphorylation of AKT (Ser473) and GSK-3β (Ser9) was measured by ELISA according to the manufacturer’s protocol (Abcam, USA), normalized to the corresponding total protein levels, and expressed as fold change relative to vehicle control.

### 4.5. Molecular Docking

The three-dimensional structure of TE2 was docked against AKT1 (PDB ID: 7NH5) and GSK-3β (PDB ID: 5K5N) using CB-Dock3 [22]. The resulting protein–ligand complexes were analyzed to identify key intermolecular interactions, and two-dimensional interaction diagrams were generated using PoseView (version 1.9; BioSolveIT GmbH, Sankt Augustin, Germany). Hydrogen bonds were defined using a donor–acceptor distance cutoff of <3.5 Å between ligand donor or acceptor atoms and the corresponding protein residue atoms.

### 4.6. Statistical Analysis

Data are presented as the mean ± standard deviation (SD) from three independent experiments. Comparisons between two groups were performed using an unpaired, two-tailed Student’s *t*-test. Comparisons among three or more groups were performed using one-way analysis of variance (ANOVA), followed by Tukey’s multiple-comparisons post hoc test. Statistical significance was defined as *p* < 0.05.

## 5. Conclusions

TE2 suppresses RANKL-induced osteoclastogenesis by modulating the AKT/GSK-3β signaling axis and reduces the viability of mature osteoclast cultures. Molecular docking predicts that TE2 may interact with both AKT1 and GSK-3β, providing a testable hypothesis for its mechanism of action. These findings identify TE2 as a promising natural-product candidate for further investigation as a modulator of pathological osteoclast formation and bone resorption.

## Figures and Tables

**Table 1:** Predicted docking scores and key residue interactions of theasaponin E2 (TE2) with AKT1 and GSK-3β.

Target	PDB ID	Binding Energy (kcal/mol)	Hydrogen-Bonding Residue	Distance (Å)	Hydrophobic Contacts
AKT1	7NH5	−9.8	Tyr18A	2.86	Lys297A, Gly311A, Cys310A
			Asp274A	2.79	
			Glu278A	2.97	
			Leu156A	3.19	
GSK-3β	5K5N	−7.7	Thr138A	2.82	Gln185A, Leu188A
			Ser203A	2.90	
			Arg220A	2.95	
			Ser66A	3.00	
			Cys218A	2.99	
			Ser219A	3.06	

Note: Binding energies are predicted docking scores obtained using CB-Dock3. Hydrogen-bond distances represent donor–acceptor distances. Protein–ligand interactions were identified using PoseView. The suffix “A” in the residue labels denotes chain A. PDB, Protein Data Bank; Å, angstrom; TE2, theasaponin E2.

## Data Availability

The original contributions presented in this study are included in the article. Further inquiries can be directed to the corresponding author.
